# Intermittent administration of parathyroid hormone improves the repairing process of rat calvaria defects: A histomorphometric and radiodensitometric study

**DOI:** 10.4317/medoral.20412

**Published:** 2015-06-02

**Authors:** Eduardo-de-Paula Silva, Daniel-Fernando-Pereira Vasconcelos, Marcelo-Rocha Marques, Marco-Antônio Dias da Silva, Flávio-Ricardo Manzi, Silvana-Pereira Barros

**Affiliations:** 1 Department of Morphology, Division of Histology, School of Dentistry at Piracicaba, University of Campinas, Piracicaba-SP, Brazil; 2 Division of Histology and Embryology, School of Biomedicine, Federal University of Piauí, Parnaíba-PI, Brazil; 3 Division of Histology and Embryology, Federal University of Campina Grande, Patos-PB, Brazil; 4 Department of Dentistry, Pontificial Catholic University of Minas Gerais, Belo Horizonte, Minas Gerais, Brazil; 5 Center for Oral and Systemic Diseases, Department of Periodontology, UNC School of Dentistry, University of North Carolina at Chapel Hill, USA

## Abstract

**Background:**

The aim of this study was to evaluate the effects of intermittent treatment of parathyroid hormone (PTH (1-34)) on the bone regeneration of critically-sized rat calvarial bone defects.

**Material and Methods:**

Thirty-two male rats were trephined (4mm fullthickness diameter), in the central part of the parietal bones and divided into 2 groups of 16. The PTH group received subcutaneous injections of PTH (1-34) at 40µg/kg, 3 times a week and the control (CTL) group received the vehicle in the same regimen. The rats were sacrificed at 4 weeks post-treatment regimen, the parietal bones were extracted and samples were evaluated through histomorphometry and radiodensitometry.

**Results:**

The histological observations showed that the PTH group presented more “island-like” new bone between the defect margins with fibrous tissues than did the CTL group. The PTH group significantly exhibited greater histologic bone formation than did the CTL group (1.5mm ±0.7; 1.9 mm ± 0.6, *p*<0.05/ for residual bone defect). The radiodensitometry analysis revealed significant differences among the PTH and CTL groups (2.1 Al eq. ±0.04; 1.8Al eq. ±0.06, *p*<0.05), demonstrating an increase in bone mineral density. The PTH treatment contributed to the bone formation with a higher amount of mineral and/or fibrous tissue when compared with the CTL group.

**Conclusions:**

The results suggest that it was possible to increase the process of bone regeneration by accelerating the healing process in rat calvarial defects through intermittent administration of the PTH treatment.

**Key words:** Bone, skull, rats, bone regeneration, bone density.

## Introduction

Defects in bone are frequent sequelae of pathological conditions such as tumors, cysts, infections, trauma or surgery. The slow regeneration of bone in such defects can pose therapeutic problems. Various strategies to stimulate healing of bone defects have been tested. These include auto grafting and allografting of bone, application of growth factors, polymeric membranes, enamel matrix derivative and others (1-3).

Various drugs have been used to assist in bone regeneration, in a way stimulate bone tissue anabolism (4,5).

We have demonstrated that the PTH intermittent treatment was able to prevent or minimize bone loss caused by induced periodontitis in rats (6,7) and also improved the healing fenestration bone defect in rat mandible (8) 

PTH functions as a major mediator of bone remodeling and as an essential regulator of calcium homeostasis, producing several distinct and independent effects on bone remodeling process, resulting in both bone formation (anabolic activity) and bone remodeling (catabolic activity), e.g. the continuous administration of PTH is characterized by large numbers of osteoclasts and enhanced osteoclast activity, rapid turnover and decrease of bone mass, while intermittent administration increases bone mass by stimulating osteoblast differentiation and prevention of osteoblast apoptosis (9).

PTH has been used successfully in the treatment of osteoporosis in humans. Intermittent administration of PTH has been proved to decrease the risk of vertebral and non-vertebral fractures and to increase the femoral and vertebral bone density with reduced or absent side effects (10). Studies have also demonstrated that the intermittent PTH treatment enhances fracture strength and callus amount in the healing of tibia fractures in rats (11), also increasing stiffness, bone density and the ultimate load in newly-formed bone after osteogenesis distraction in rats (12). Although most of these studies support a role for PTH in the treatment of osteoporosis and long bone fractures, only a few studies associated PTH treatment to calvarial healing (13,14).

Andreassen and Cacciafesta (14) reported that intermittent PTH administration enhanced mechanical strength of healing rat calvarial defects covered by expanded polytetrafluoroethylene membranes, besides having increased the dry weight of the newly-formed tissue inside the defect. Stancoven *et al*. (13) used a critical-size of 8mm and investigated the potential of recombinant human bone morphogenetic protein-2 soak-loaded onto an absorbable collagen sponge to induce local bone formation compared with the clinical reference demineralized bone matrix and investigated potential additive/synergistic effects of exogenous PTH.

The purpose of our study was to evaluate the effect of intermittent PTH treatment alone on critically-sized calvarial defects (4mm) in Wistar rats through histomorphometric and radiodensitometric analyses.

## Material and Methods

- Animals 

Thirty-two male Wistar rats, weighing an average of 250 g, were used in this study. The animals were separated randomly into two groups (16 rats per group) and kept in cages in an animal room having a photo period of 12 h of light with access to industrial food, close to 110 Kcal/kg of body weight; water was provided “ad libitum.” Room temperature was kept at between 22-25°C, and animals were allowed one week to adapt to the environment. The University of Campinas Institutional Animal Care and Use Committee approved the protocol under number 737-2/ UNICAMP-CEEA-IB-UNICAMP.

- Surgical Technique

The animals were anesthetized with intramuscular administration of a solution of 13 mg/Kg of 2% xylazine hydrochloride (Rompum-Bayer-São Paulo, SP, Brazil) and 33 mg/Kg of ketamine (Francotar-Virbac-Roseira, SP, Brazil). During surgery, local anesthesia (lidocaine, 1:100.000 epinephine) was used at the surgical site to reduce local bleeding.

The surgical site was trichotomized and scrubbed with iodine. An incision approximately 20mm long was made in the scalp along the sagittal suture and full-thickness flap, including periosteum, was reflected, exposing the calvarial bone. Afterwards, two transosseous defects standardized with 4mm each in diameter were created in the center of the parietal bone without damaging the dura mater. A 4mm trephine bur (Neodent, Curitiba, PR, Brazil) was used to create the defects under constant irrigation with sterile physiological solution, to prevent the overheating of bone margins. The periosteum and skin were then sutured for total coverage using 4-0 nylon point sutures (Ethicon, Johnson & Johnson, São José dos Campos, SP, Brazil).

**Figure 1:**
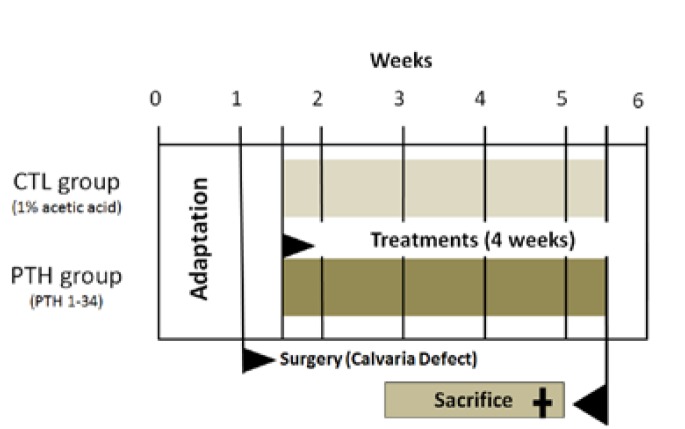
Animal Experiments demonstrate the adaptation time, beginning treatments (PTH and CTL groups), surgery (Calvaria defects), and the time of sacrifice.

Three days after surgery, the animals were divided into 2 groups of 16 animals each. To the experimental group, PTH group, 40µg/kg of PTH was given (1-34) (Sigma-Aldrich, St. Louis, MO) prepared in 1% acetic acid, injected subcutaneously, 3 times a week for 4 weeks. The control group (CTL group) received the same volume of the vehicle according to figure 1. The intermittent PTH schedule and dose used in the present study were based on previous study (6-8). Neither antibiotics nor anti-inflammatory drugs were administered after the surgery. Acetaminophen (Paracetamol, Abbott Laboratories, São Paulo, São Paulo, Brazil) was used for pain control.

- Radiographic Procedures

After 4 weeks of treatment, the animals were sacrificed through overdose of anesthetics. Sections of bone tissue, including the surgical sites, were removed with 701bur (Neodent, Curitiba, PR, Brazil) under constant irrigation and fixed in 4% neutral formalin solution for 72h. All samples were radio graphed using a dental x-ray unit (GE-1000) with an exposure time of 0.1 seconds and 31x41mm dental radiographic film (Insight Film, Eastman Kodak, Rochester, NY) and radiographs were densitometrically measured through aluminum step-wedge equivalent thickness (Al eq.). The mean values of an optical density were obtained from 5 measurements of the defect area.

- Histological Procedures

**Figure 2:**
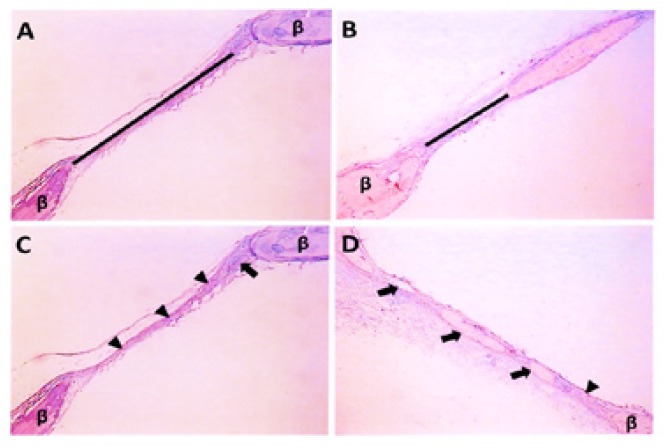
Photomicrographs illustrating the section of critically-sized defect in calvaria of Wistar rat from different groups stained with hematoxylin and eosin. A) Black line indicates residual bone defect measured in CTL group (original magnification 35x). B) Black line indicates residual bone defect measured in PTH group (original magnification 35x). C) Arrowheads indicate fibrous tissue and arrow indicates island-like of new bone in the CTL group (original magnification 35x). D) Arrowheads indicate fibrous tissue and arrows indicate island-like of new bone in the CTL group (original magnification 25x). β = Bone.

After the radiographic procedures, all samples were demineralized in a 5% EDTA/phosphate buffered saline solution for 60 days and embedded in paraffin (15). Five-µm thick sections including the center of circular defects were stained with Hematoxylin and Eosin. Paraffin serial sections were measured using an image analysis system (Image-Pro, Media Cybernetics, Silver Spring, MD and Program KS400, Kotron Elektron GmbH, Eching, Germany). The residual bone defect was defined as the distance of the bone margins after the treatment period (Fig. 2A). In the presence of areas of new bone formation between the margins, the total measurement was calculated deducting the new bone island-like formations (Fig. 2B).

- Statistical Analysis

Student’s t test was used for the comparison between experimental and control groups. The data were submitted to statistical analysis and p values lower than 0.05 were considered significant (BioEstat ver.5.0, Belém, PA, Brazil).

## Results

- Clinical observations

No adverse events were observed during the healing interval. After 4 weeks, the defects in PTH group were smaller and the edges were more irregular than in the CTL group. Bone regeneration occurred in form of islands-like, more evident characteristics in the PTH group (Fig. 2D) compared to the CTL group (Fig. 2C). Neither of the specimens exhibited osseous continuity in both edges of the defect after treatment for 4 weeks.

- Histological Findings

**Figure 3:**
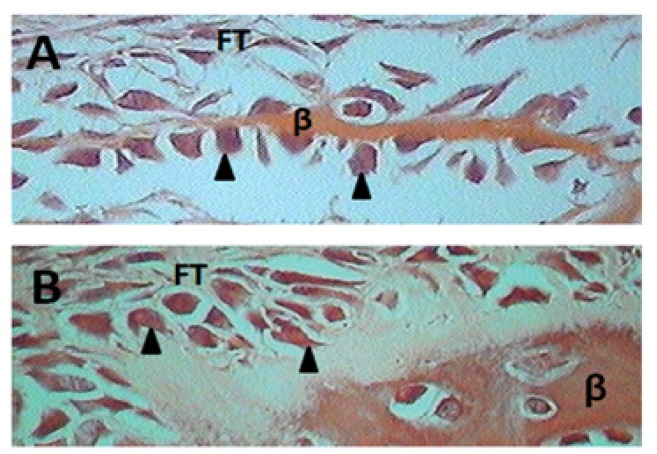
Photomicrographs of defect in calvaria of Wistar rat from different groups stained with hematoxylin and eosin, original magnification 600x. Histological details of the CTL group in A and PTH group in B. Arrowheads indicate osteoblasts, FT = fibrous tissue, β = Bone.

The histological observations through light microscopy showed that in the PTH group, the bone defects were filled with new bone formation and thick fibrous tissue (Fig. 2B, D and Fig. 3B). In comparison, the CTL group showed that the bone defect was filled mainly with fibrous tissue (Fig. 3A) and a little island-like new bone formation between the margins of the initial defect (Fig. 2C).

Bone Histomorphometric Analysis

The histomorphometric analysis of the residual defect showed that samples from the PTH group presented mean of 1.5mm ±0.7 in diameter; for the CTL group, the mean diameter was 1.98mm ±0.6 at 4 weeks post-surgery. There were statistically significant inter group differences (*p*< 0.05).

Radiodensitometric Analysis

Statistical analysis (*p*<0.05) of optical density revealed significant differences among the PTH group (2.1Al eq. ±0.04) and the CTL group (1.8Al eq. ±0.06).

## Discussion

Recombinant human PTH (1-34) has been approved in the United States as mono therapy for the treatment of post menopausal women with osteoporosis. Since PTH has been approved for the treatment of osteoporosis, a number of questions have arisen and PTH, in combination with other antiresorptive agents, has also been used for the treatment of osteoporosis (16-18). However, clinical administration of PTH alone is a potential therapy that has been shown to enhance and accelerate bone formation in bone fractures, regions surrounding dental implants (19,20) and in fenestrated mandibular defect (8).

The critic ally-sized bone defect has been defined as the size of an osseous defect that does not heal spontaneously with bone during the lifetime of the animal (21). Aalami *et al*. (22) demonstrated that the full-thickness 3, 4 and 5mm diameter in mice´s calvarial defect is not completely healed after 8 weeks.

In this study, we used a critic ally-sized 4mm calvarial defect in rats that were ten weeks old in the begging of the experiment. The treatment with intermittent PTH administration was given for 4 weeks and as expected, in both groups, PTH and CTL, there was an incomplete healing of the defects in this period studied. However, it was possible to observe the presence of new bone formation as islands-like between the margins of the initial 4mm defect.

It has been demonstrated that with the PTH intermittent treatment, it was possibly to prevent bone loss and restore bone mass in ovariectomized rats (23). Shirota *et al*. (20) evaluated the influence of intermittent PTH (1-34) administration on the healing process after the placement of titanium screw implants in ovariectomized rats. The authors found that the density of bone around implants in the PTH group was almost the same found in the Sham operated group after 56 days of implantation, suggesting that the PTH treatment might be useful to improve clinical results after dental implantation.

Our previous work showed that the PTH (1-34) treatment using 40 µg/kg, 3 times a week, was able to protect the tooth site from periodontitis-induced bone loss and promoted significant reduction in the number of inflammatory cells at the marginal gingival area in ovariectomized rats (6,7).

Another investigation of our research team (8) demonstrated the PTH (1-34) administration was effective to induce bone formation, increase bone density, increase the amount of callus formed and also to accelerate the callus reabsorption, an increase in the number of osteoclasts present in the callus region, corroborating with other studies (9,12-14,24).

We also investigated the bone mineral density (BMD) that is widely used to evaluate alterations in the balance between osteoblastic bone formation and osteoclastic bone resorption (25). The measurement of BMD through radiographic densitometry has been performed in a number of studies, and its efficacy has been confirmed (25-27). Radiographic densitometry analysis is an important tool for the research evaluation because it is a rapid and low-cost method, and can produce results which are similar to those of dual-energy x-ray absorptiometry (25).

Our study was the first to use radiographic densitometry for the analysis of the intermittent PTH treatment in calvarial defect. We demonstrated that the PTH (1-34) intermittent treatment increased the BMD in rat calvaria critically-sized defect model. These results reinforce the histological observations due to, possibly a speculation, bone collagen during the mineralization phase, and changes in bone turnover could be expected to affect the quality of the bone, corroborating in a similar way with our previous study observed in the characteristics of collagen fibers from the periodontal ligament visualized through polarizing light microscopy (8). Garnero *et al*. (28) demonstrated that the PTH treatment changed type-I collagen isomerization (28).

Stancoven *et al*. (13) demonstrated that with PTH (1-34) administered (15 µg/kg) daily on calvarial defect (8mm) analyzed through radiographic and histologic exams, bone formation was slightly increased in the PTH group without a membrane and in the PTH group with a membrane. Andreassen and Cacciafesta (14) reported that daily PTH (1-34) administration (60µg/kg) for 5 weeks enhanced mechanical strength of healing rat´s calvarial defects (5mm) covered with expanded polytetrafluoroethylene membranes, and increased the dry weight of the newly-formed tissue inside the defect.

We demonstrated that treatment with a lower frequency of PTH injections, 3 times a week, for 4 weeks using dose PTH at 40µg/kg, was able to contribute to the acceleration of the healing process in the absence of some type of membrane to isolate the defect (4mm). We also demonstrated an increase in optical density measurements (bone mineral density) in the PTH group; samples suggested that PTH treatment contributed to bone formation with a higher amount of mineral and/or fibrous tissue when compared with the CTL group. These results were associated with the frequent observation of “island-like” format of new bone in the PTH group and a possible organization of the new collagen fibers, as previously demonstrated (8,28).

In conclusion, the present study reinforced the anabolic potential of PTH (1-34) intermittent treatment alone, without the utilization of any coadjuvant, suggesting that it is possible to increase the process of bone regeneration by accelerating the healing process in rat´s calvarial defects.
